# LncRNA ALKBH3‐AS1 enhances ALKBH3 mRNA stability to promote hepatocellular carcinoma cell proliferation and invasion

**DOI:** 10.1111/jcmm.17558

**Published:** 2022-09-13

**Authors:** Qiliang Lu, Hao Wang, Xiangxiang Lei, Qiancheng Ma, Jie Zhao, Wen Sun, Cheng Guo, Dongsheng Huang, Qiuran Xu

**Affiliations:** ^1^ Qingdao Medical College Qingdao University Qingdao China; ^2^ The Key Laboratory of Tumor Molecular Diagnosis and Individualized Medicine of Zhejiang Province, Zhejiang Provincial People's Hospital Affiliated People's Hospital, Hangzhou Medical College Hangzhou China; ^3^ Department of Hepatobiliary Surgery The First Affiliated Hospital of Xi'an Jiaotong University Xi'an China; ^4^ Hangzhou Medical College Hangzhou China; ^5^ Zhejiang University of Technology Hangzhou China; ^6^ The Second Clinical Medical College Zhejiang Chinese Medical University Hangzhou China

**Keywords:** ALKBH3, ALKBH3‐AS1, hepatocellular carcinoma, hypoxia, mRNA stability

## Abstract

Long noncoding RNAs (lncRNAs) are confirmed as the key regulators of hepatocellular carcinoma (HCC) occurrence and progression, but the role of AlkB homologue 3 antisense RNA 1 (ALKBH3‐AS1) in HCC is unclear. We revealed the overexpression of ALKBH3‐AS1 in HCC tissues. The upregulated levels of ALKBH3‐AS1 were observed in HCC cells. ALKBH3‐AS1 was expressed in the nucleus and cytoplasm of HCC cells. The high ALKBH3‐AS1 expression was markedly associated with a decreased survival rate of HCC patients. ALKBH3‐AS1 knockdown repressed and ALKBH3‐AS1 overexpression enhanced HCC cell invasion and proliferation. ALKBH3‐AS1 silencing restricted HCC growth in vivo. A significant positive correlation between ALKBH3‐AS1 and ALKBH3 mRNA levels was confirmed in HCC specimens. ALKBH3‐AS1 silencing reduced ALKBH3 expression by stabilizing its mRNA stability in HCC cells. Notably, the impact of ALKBH3 silencing on HCC cells was similar to that of ALKBH3‐AS1 knockdown. ALKBH3 restoration prominently attenuated the suppressive effects resulting from ALKBH3‐AS1 silencing in HCCLM3 cells. Hypoxia‐inducible factor‐1α (HIF‐1α) transcriptionally activated ALKBH3‐AS1 expression in hypoxic HCC cells. ALKBH3‐AS1 knockdown markedly attenuated cell proliferation and invasion in hypoxic Huh7 cells. Collectively, HIF‐1α‐activated ALKBH3‐AS1 exerted an oncogenic role by enhancing ALKBH3 mRNA stability in HCC cells.

## 1 INTRODUCTION

The World Health Organization's International Agency for Research on Cancer (IARC) released the latest global cancer burden data in 2020.^1^ The number of new cases of liver cancer, 90% of which is hepatocellular carcinoma (HCC), ranks 6th among all tumours, and it ranks 3rd in the number of deaths.^1^ Due to the lack of specific biomarkers, the advanced HCCs were diagnosed in most patients, which lose the opportunity to receive radical treatment.^2^ Many patients with a high risk of recurrence of HCC also do not receive close postoperative monitoring and timely treatment.^2^ In addition, the molecular mechanisms of HCC growth, metastasis, immune escape and treatment resistance are complex, which is a hot and challenging point of current research.

Long noncoding RNA (lncRNAs) are defined as transcripts longer than 200 nucleotides that lack protein‐coding capacity, and so far, more than 10,000 lncRNAs have been identified in the human genome.^3^ LncRNAs are important modulators of gene expression at different levels, such as chromatin remodelling, transcription and post‐transcriptional processing.^4^ Notably, lncRNAs have been confirmed as diagnostic and prognostic biomarkers and the critical regulators of HCC occurrence and progression.^4^, ^5^, ^6^ LncRNA p53‐stabilizing and activating RNA (PSTAR) is a new tumour‐suppressive factor in HCC. PSTAR enhances the SUMOylation of heterogeneous nuclear ribonucleoprotein K (hnRNP K) and promotes its interaction with p53, thereby leading to p53 transactivation and repressing HCC tumorigenicity.^7^ LncRNA NIHCOLE expression is downregulated in HCC and confers to poor clinical outcomes for patients.^8^ NIHCOLE induces apoptosis and suppresses HCC cell proliferation by leading to the nonhomologous end‐joining (NHEJ) pathway inactivation.^8^ Our research team has done a lot of work on the roles and regulatory mechanisms of lncRNAs in HCC occurrence and development.^9^, ^10^, ^11^, ^12^, ^13^, ^14^, ^15^ For instance, we demonstrate that lncRNA minichromosome maintenance complex component 3 associated protein antisense RNA 1 (MCM3AP‐AS1) is frequently overexpressed in HCC and confers poor clinical outcomes.^9^ MCM3AP‐AS1 upregulates forkhead box A1 (FOXA1) by sponging miR‐94‐5p and then facilitates HCC growth.^9^ Hypoxia‐inducible factor‐1α (HIF‐1α)‐activated transmembrane 4 L six family member 1 antisense RNA 1 (TM4SF1‐AS1) enhances HCC cell migration, proliferation and invasion via increasing TM4SF1 expression.^11^ LncRNA P38 inhibited cutaneous squamous cell carcinoma‐associated lincRNA (PICSAR) is overexpression and significantly confers poor prognosis of HCC.^15^ PICSAR facilitates HCC progression by competitively binding miR‐588 and accordingly enhancing eukaryotic translation initiation factor 6 (EIF6)‐mediated phosphatidylinositol 3‐kinase (PI3K)/AKT/mechanistic target of rapamycin kinase (mTOR) pathway.^15^ AlkB homologue 3 antisense RNA 1 (ALKBH3‐AS1) is located on human chromosome 11p11.2 and is a lncRNA of about 1927 nucleotides in length. So far, the expression and role of ALKBH3‐AS1 in HCC remain unclear.

In our study, the expression difference and clinical significance of ALKBH3‐AS1 in HCC were analysed. We investigated the biological functions of ALKBH3‐AS1 and its underlying mechanisms in HCC cells. Finally, the impact of hypoxia on ALKBH3‐AS1 was investigated. We demonstrated that HIF‐1α‐activated ALKBH3‐AS1 exerted an oncogenic role by enhancing ALKBH3 mRNA stability in HCC cells.

## 2 MATERIALS AND METHODS

### 2.1 Tissue samples

This study collected tumour and paired nontumor tissue samples from 80 patients with HCC after obtaining written informed consent in the First Affiliated Hospital of Xi'an Jiaotong University. Inclusion criteria were as follows: (1) All patients were pathologically diagnosed with HCC; (2) all of the patients did not receive treatment before surgery; (3) all patients underwent R0 surgical resection; (4) all patients had complete medical records and follow‐up data. Exclusion criteria were as follows: (1) Combined with other malignant tumours; (2) the patient developed other malignancies during follow‐up. All the collected tissue samples were well preserved at −80°C. Our study was checked and approved by the Ethics Committee of the 1st Affiliated Hospital of Xi'an Jiaotong University (No: XJTU1AF2021LSK‐135). The clinicopathologic parameters of HCC patients are shown in Table S1.

### 2.2 Cell culture

Human normal hepatocytes (MIHA) were provided by bnbio (Beijing, China). HepG2, Huh7, Hep3B, MHCC97H and HCCLM3 cells were previously provided by Stem Cell Bank, Chinese Academy of Sciences (Shanghai, China). The above cell lines were cultured in previously described conditions.^16^

### 2.3 Lentivirus and plasmids

LKO.1‐puro lentiviral vectors containing shRNA against ALKBH3‐AS1 (shAS1#1 and shAS1#2), ALKHB3 (shALKBH3), HIF‐1α (shHIF1A) and non‐targeting shRNA (NTC) were purchased from GeneChem (Shanghai, China). The packaging and infection of lentivirus were carried out as previously described.^11^ The full‐length of ALKBH3‐AS1 and ALKBH3 cDNA were sub‐cloned into pcDNA3.1 (Invitrogen, Carlsbad, CA, USA), respectively. Lipofectamine 3000 (Thermo Fisher Scientific, Waltham, MA, USA) was applied for plasmid transfection in HCC cells.

### 2.4 Real‐time quantitative PCR (RT‐qPCR)

The Trizol reagent (Invitrogen) was applied for RNA extraction. Then, the RNA was reversely transcribed into cDNA using PrimeScript™ RT Master Mix (Takara, Shiga, Japan), following the manufacturer's protocol. RT‐qPCR was conducted using SYBR Green PCR Master Mix (Takara) in the CFX96 Touch™ real‐time PCR detection system (Bio‐Rad Laboratories, Hercules, CA, USA). The relative mRNA expression was normalized to β‐actin according to the 2^−ΔΔct^ calculation method. ALKBH3‐AS1: 5′‐TCC AGA GTC CCT GAC GCA TA‐3′ (forward) and 5′‐AAG GCT TTC CTG ATG CCT CC‐3′ (reverse); ALKBH3: 5′‐TAC CAC TGC TAA GAG CCA TCT CC‐3′ (forward) and 5′‐GAC AGG CTG ATT TCA TAC ACA CC‐3′ (reverse); β‐actin: 5′‐TGA CCC AGA TCA TGT TTG AG‐3′ (forward) and 5′‐CGT ACA GGG ATA GCA CAG‐3′ (reverse).

### 2.5 Subcellular fractionation detection

The RNA was, respectively, isolated from the nucleus and cytoplasm of HCC cells following the protocol of the PARIS™ kit (Thermo Fisher Scientific). The expression level of ALKBH3‐AS1 in the nucleus and cytoplasm of HCC cells was detected by RT‐qPCR.

### 2.6 Fluorescence in situ hybridization (FISH) analysis

Cy3‐labelled ALKBH3‐AS1 probe was designed and produced by RiboBio (Guangzhou, China). Subcellular localization of ALKBH3‐AS1 was analysed by the FISH Kit (RiboBio).

### 2.7 Western blotting

HCC cells were scraped with RIPA buffer (Beyotime, Shanghai, China) and lysed on ice for 20 min, followed by centrifugation. For concentration determination, the lysate supernatant was collected and subjected to Enhanced BCA Protein Assay Kit (Beyotime). Protein samples (20 μg per lane) were separated on 10% SDS‐PAGE gel. After protein transfer, the PVDF membrane (Millipore, Billerica, MA, USA) was blocked with 5% Milk/TBST and incubated with the appropriate primary antibodies, including HIF‐1α (ab1, Abcam, Cambridge, MA, USA), ALKBH3 (ab227496, Abcam) and β‐tubulin (10094‐1‐AP, Proteintech, Wuhan, China), overnight at 4°C. The membrane was washed thrice with TBST followed by incubation with the HRP‐conjugated secondary antibodies (Beyotime) for 2 h. The washed membrane was eventually subjected to image capture using the Amersham Imager 680 (GE Healthcare Life Sciences, Pittsburgh, PA, USA) in the presence of Immobilon Forte Western HRP Substrate (Millipore).

### 2.8 Cell proliferation and invasion assays

Cell invasion and proliferation potentials were determined by transwell chamber, CCK‐8 kit (Beyotime) and Cell‐Light™ EdU Apollo®488 In Vitro Imaging Kit (RIBOBIO, Guangzhou, China), respectively.^9^, ^11^

### 2.9 In vivo experiments

Eight male nude mice (4–5 weeks) were randomly divided into two groups, and 5 × 10^6^ HCCLM3 cells stably transfected with NTC or shAS1#1 were injected into the left upper limb of the mice. Tumour volumes were measured once a week. Four weeks later, cervical dislocation killed nude mice to obtain tumours. Tumour weight was measured and calculated, and 4‐week tumour growth curves were drawn. The xenograft tumours were subjected to RT‐qPCR for ALKBH3‐AS1 expression. Ki‐67 staining was carried out using the corresponding antibody (27309‐1‐AP, Proteintech) in tumour tissues. Animal research was approved by the Institutional Animal Care and Use Committee of Xi'an Jiaotong University.

### 2.10 Nascent RNA capture analysis

According to the product's instructions, nascent RNA was isolated with the Click‐iT Nascent RNA Capture Kit (Thermo Fisher Scientific) for RT‐qPCR analysis as previously described.^17^

### 2.11 Statistical analysis

GraphPad Prism 8 (San Diego, CA, USA) was used for statistical analyses. The above in vitro assays were all repeated three times. The Mann–Whitney test, Student's *t*‐test, chi‐square test and anova were used for data analysis. Data are expressed as mean ± SD. *p* < 0.05 is considered statistically significant.

## 3 RESULTS

### 3.1 The upregulated level of ALKBH3‐AS1 predicts the poor prognosis of HCC

**FIGURE 1:**
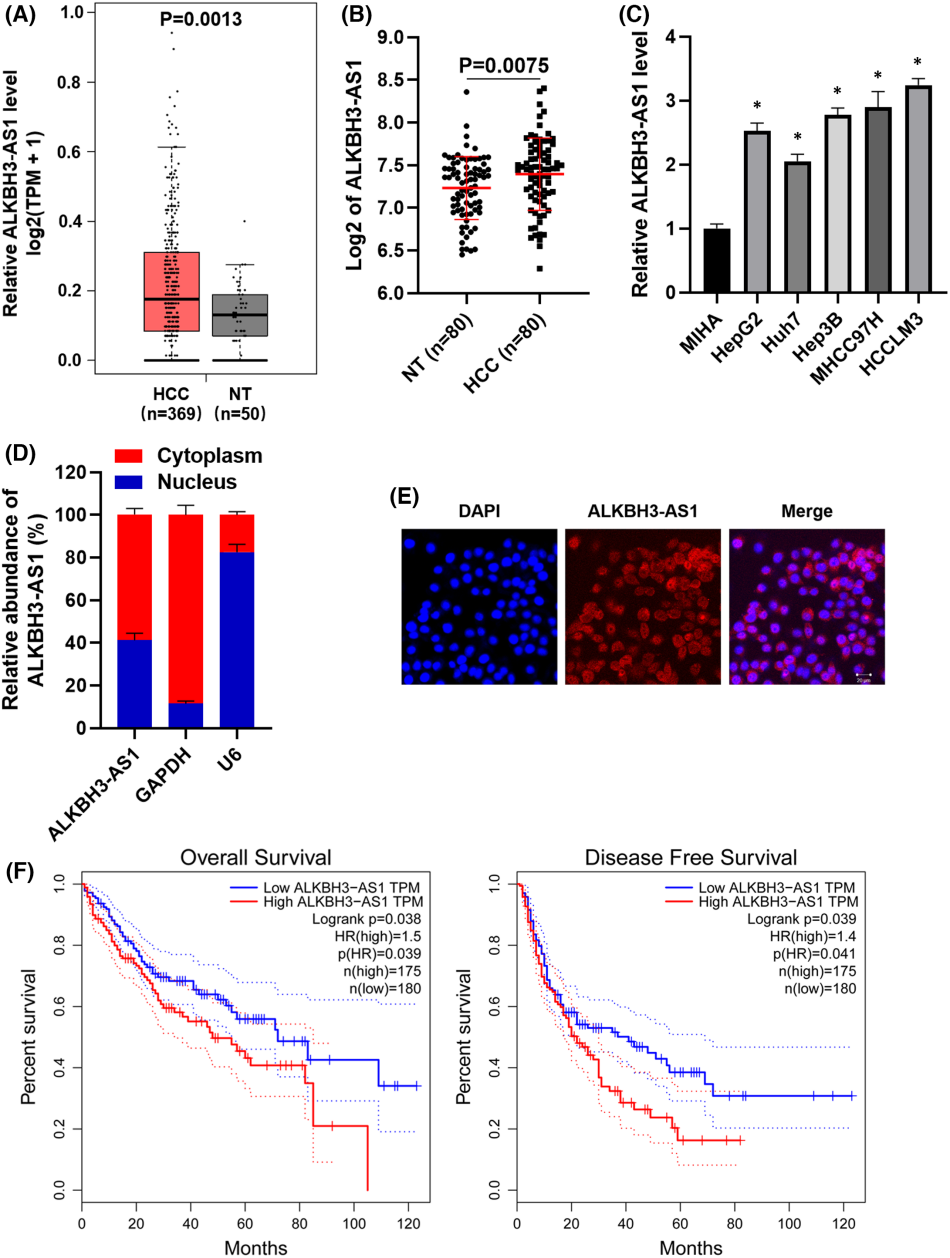
Prognostic value of ALKBH3‐AS1 expression in HCC. (A) The comparison of ALKBH3‐AS1 levels between HCC and normal liver tissues in the TCGA database was analysed on the GEPIA platform. (B) The difference of ALKBH3‐AS1 expression between HCC and adjacent nontumor tissues. (C) The levels of ALKBH3‐AS1 in MIHA, HepG2, Hep3B, Huh7, MHCC97H and HCCLM3 cells. (D) Subcellular fractionation analysis and (E) FISH assay indicated that ALKBH3‐AS1 was expressed in both the nucleus and cytoplasm of HCCLM3 cells. (F) TCGA‐LIHC data analysed by the GEPIA platform suggested that the high ALKBH3‐AS1 level indicated poorer overall survival and disease‐free survival of HCC patients. *p < 0.05

TCGA‐LIHC data analysis using the GEPIA platform^18^ revealed that the ALKBH3‐AS1 expression was markedly higher in HCC than in liver tissues (*p* = 0.0013, Figure 1A). RT‐qPCR analyses of clinical tissue samples also demonstrated that the ALKBH3‐AS1 level was prominently upregulated in HCC specimens (*p* = 0.0075, Figure 1B). The increased levels of ALKBH3‐AS1 were confirmed in HCC cell lines, including HepG2, Huh7, Hep3B, MHCC97H and HCCLM3 (*p* < 0.05, Figure 1C). Subcellular fractionation detection and FISH assay consistently demonstrated that ALKBH3‐AS1 was expressed in both the nucleus and cytoplasm of HCCLM3 cells (Figure 1D,E). As presented in Table S1, clinical analysis clarified that ALKBH3‐AS1 was expressed at higher levels in HCC samples from patients with large tumours (≥5 cm, *p* = 0.011), venous infiltration (*p* = 0.007) and advanced tumour stages (III + IV, *p* = 0.006). Moreover, survival analysis of TCGA‐LIHC data using the GEPIA platform^18^ confirmed that the higher level of ALKBH3‐AS1 predicts the poorer survival of HCC (*p* < 0.05, Figure 1F). These data suggest ALKBH3‐AS1 as a potential poor prognostic marker of HCC.

### 3.2 ALKBH3‐AS1 contributes to HCC progression

**FIGURE 2:**
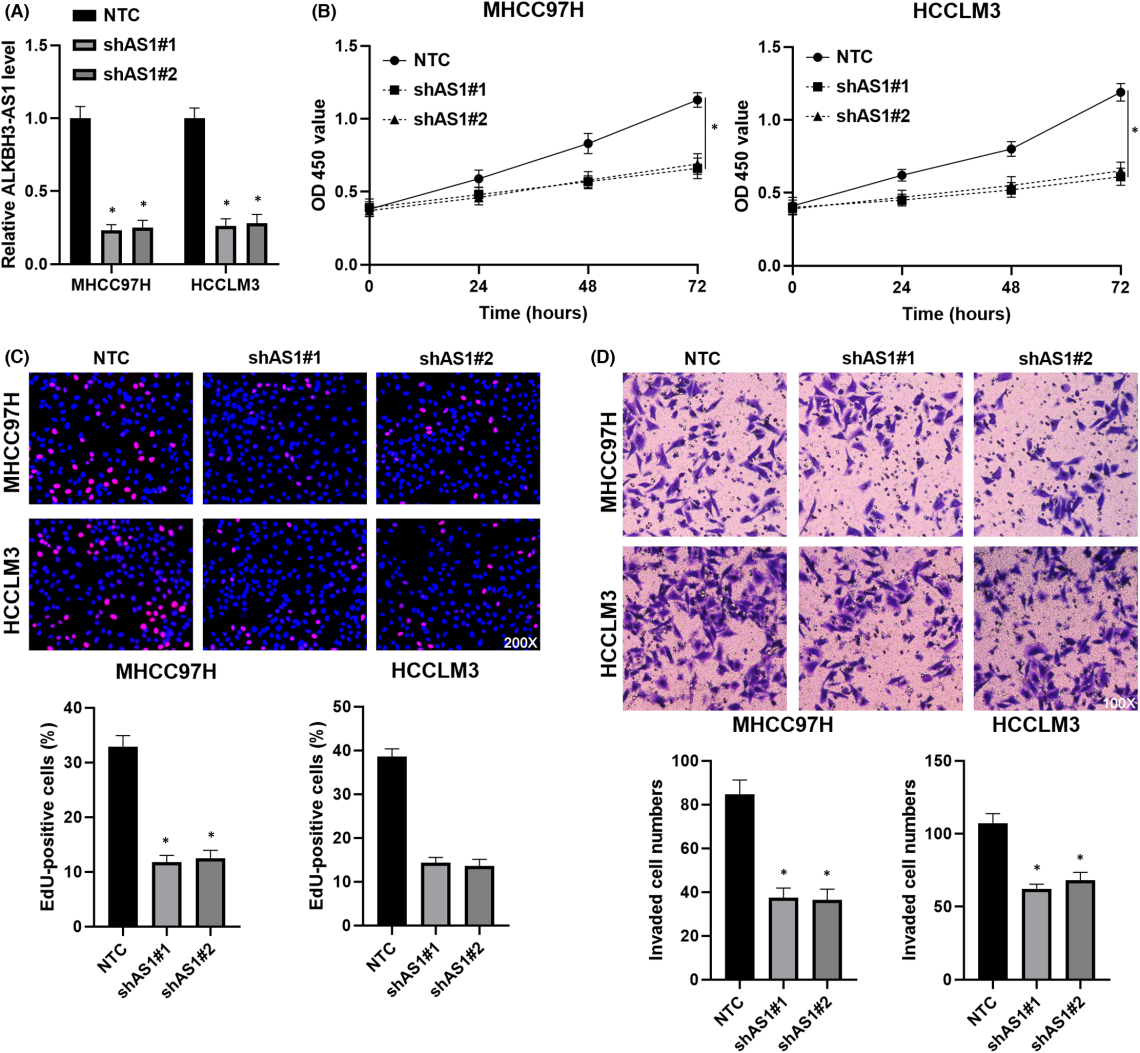
ALKBH3‐AS1 knockdown suppresses the proliferation and invasion of HCC cells. (A) ALKBH3‐AS1 shRNAs (shAS1#1 and shAS1#2) and non‐targeting shRNA (NTC) were transfected into MHCC97H and HCCLM3 cells. The ALKBH3‐AS1 level was confirmed by RT‐qPCR. (B) The cell viability was analysed in HCC cells with or without ALKBH3‐AS1 silencing using the CCK‐8 assay. (C) The percentage of Ki‐67‐positive HCC cells was decreased by ALKBH3‐AS1 knockdown. (D) The influence of ALKBH3‐AS1 knockdown in HCC cell invasion was confirmed using transwell chambers. *p < 0.05

**FIGURE 3:**
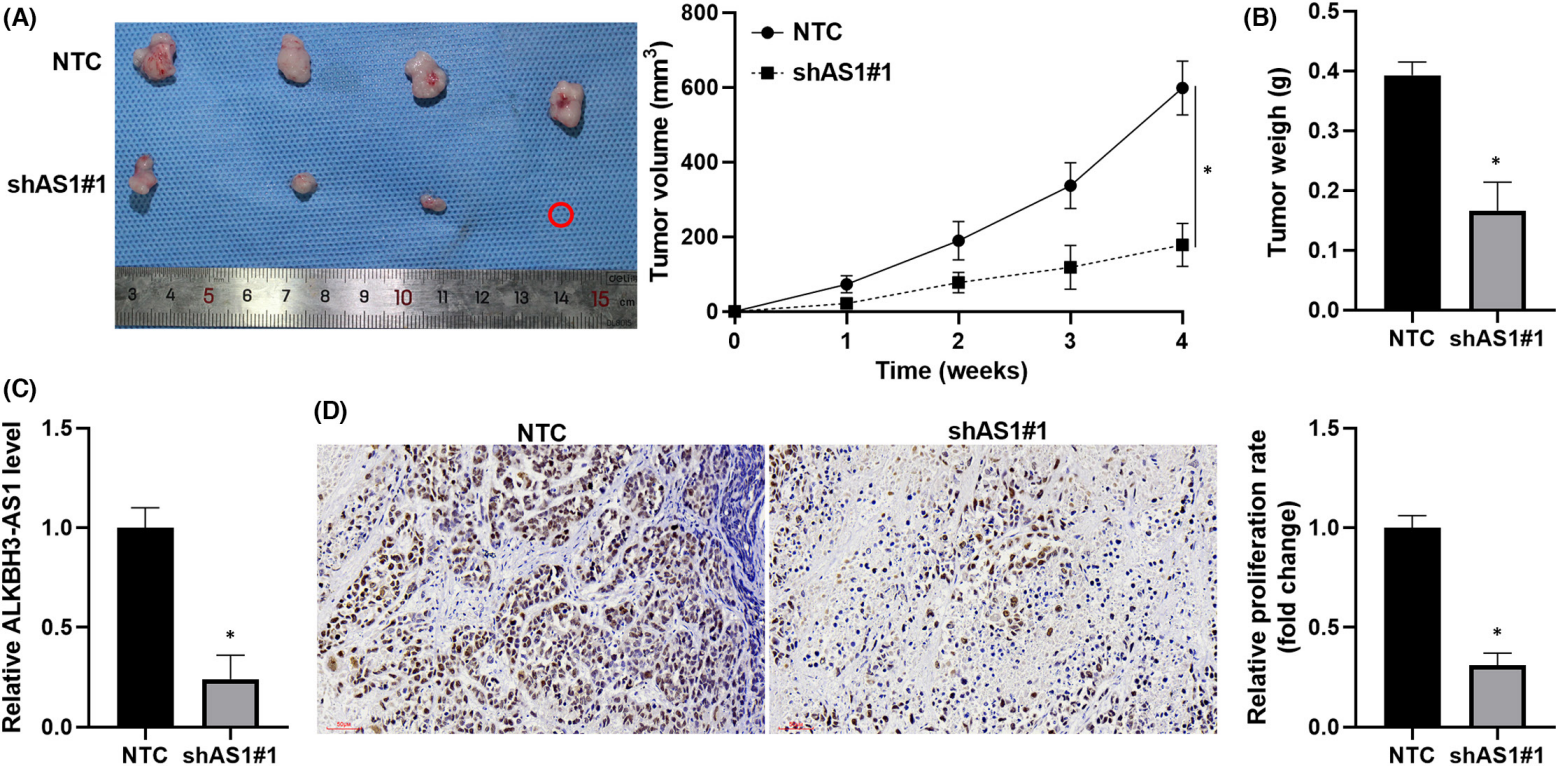
ALKBH3‐AS1 depletion restricts HCC growth in vivo. (A) ALKBH3‐AS1 knockdown and control HCCLM3 cells were, respectively, injected into male nude mice (*n* = 4). The tumour growth curves were drawn for 4 weeks. (B) The tumour weights were compared between the ALKBH3‐AS1 knockdown and control groups. (C) ALKBH3‐AS1 level was determined in tumour tissues obtained from the ALKBH3‐AS1 knockdown and control groups. (D) The Ki‐67 staining was performed in tumour tissues collected from the ALKBH3‐AS1 knockdown and control groups. *p < 0.05

ALKBH3‐AS1 knockdown was carried out in HCCLM3 and MHCC97H cells (*p* < 0.05, Figure 2A). We found that ALKBH3‐AS1 knockdown significantly weakened the viability of HCC cells (*p* < 0.05, Figure 2B). The depletion of ALKBH3‐AS1 prominently decreased HCC cell proliferation with reduced EdU staining cells (*p* < 0.05, Figure 2C). The cell invasion potential was remarkably repressed by ALKBH3‐AS1 silencing in HCC cells (*p* < 0.05, Figure 2D). Meanwhile, ectopic expression of ALKBH3‐AS1 evidently promoted the invasion and proliferation of Huh7 cells (*p* < 0.05, Figure S1). Next, ALKBH3‐AS1 knockdown remarkably decreased xenograft tumour volumes and weights in vivo (*p* < 0.05, Figure 3A,B). RT‐qPCR assay confirmed the reduced level of ALKBH3‐AS1 in tumour tissues collected from the ALKBH3‐AS1 knockdown group (*p* < 0.05, Figure 3D). The tumour samples in the ALKBH3‐AS1 knockdown group also showed a decreased percentage of Ki‐67‐positive cells than the control group (*p* < 0.05, Figure 3E). Collectively, we recognize ALKBH3‐AS1 as an oncogene in HCC.

### 3.3 ALKBH3‐AS1 enhances the stability of ALKBH3 mRNA

**FIGURE 4:**
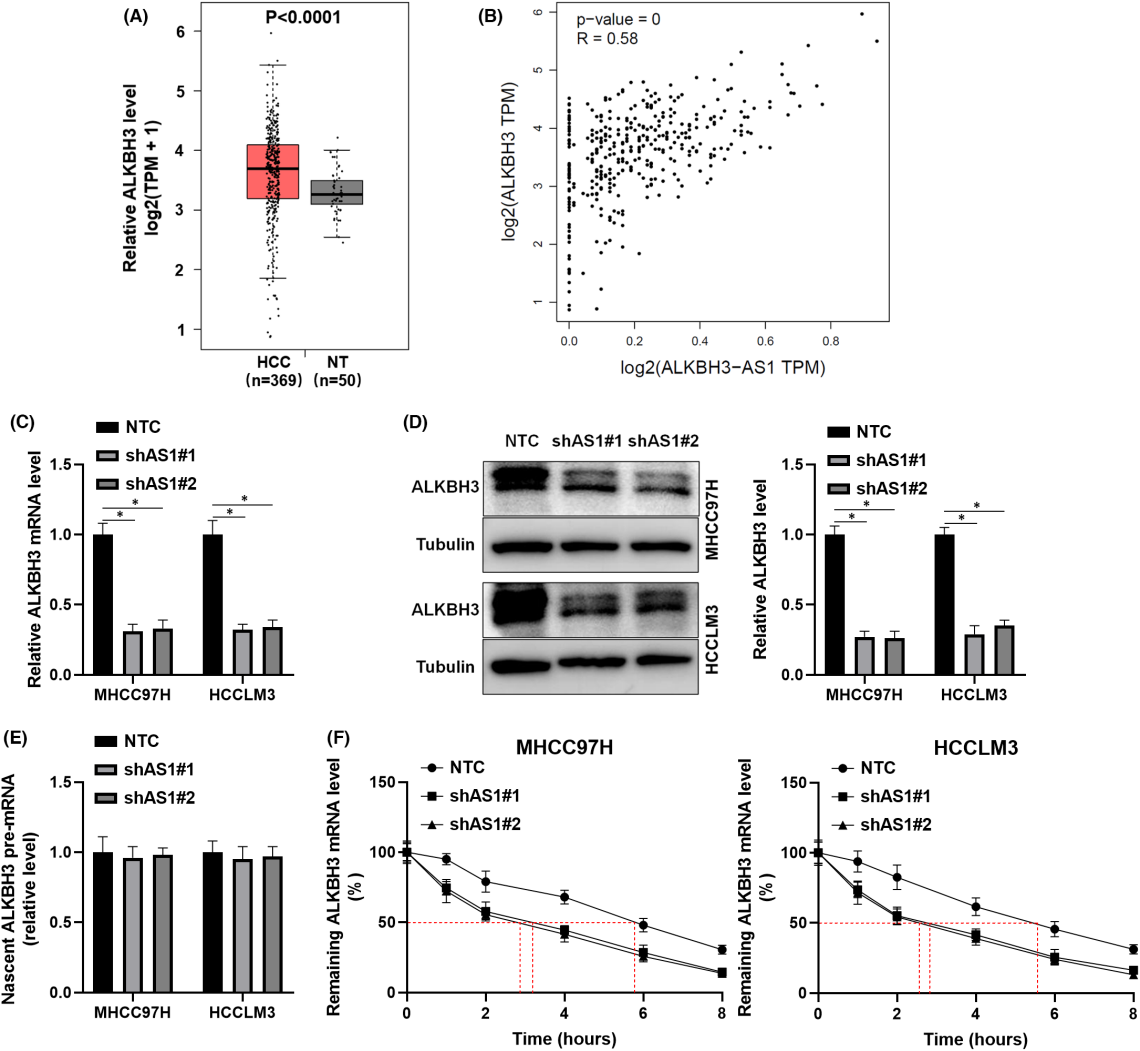
ALKBH3‐AS1 positively regulates ALKBH3 mRNA stability. (A) The comparison of ALKBH3 mRNA levels between HCC and normal liver tissues in the TCGA database was analysed on the GEPIA platform. (B) The interaction between ALKBH3‐AS1 and ALKBH3 mRNA levels was analysed in HCC tissues. (C) ALKBH3 mRNA level was determined in ALKBH3‐AS1 knockdown and control HCC cells. (D) ALKBH3 protein level was assessed in ALKBH3‐AS1 knockdown and control HCC cells. (E) RT‐qPCR confirmed the levels of nascent ALKBH3 pre‐mRNA in ALKBH3‐AS1‐silenced and control cells. (F) ALKBH3 mRNA stability was investigated in ALKBH3‐AS1‐silenced and control HCC cells with actinomycin D (50 ng/ml) treatment for different time points. *p < 0.05

Previous evidence demonstrates that antisense lncRNAs modulate their neighbouring genes' expression.^19^ Therefore, we explored the regulatory role of ALKBH3‐AS1 in ALKBH3 expression. TCGA‐LIHC data analysis using GEPIA platform^18^ found that ALKBH3 mRNA expression was upregulated and positively correlated with ALKBH3‐AS1 level in HCC tissues (*p* < 0.0001, Figure 4A,B). ALKBH3‐AS1 silencing prominently reduced ALKBH3 levels in HCC cells (*p* < 0.05, Figure 4C,D). Moreover, xenograft tumour tissues from the ALKBH3‐AS1 knockdown group expressed a significantly lower level of ALKBH3 mRNA than in the control group (*p* < 0.05, Figure S2). Mechanistically, ALKBH3‐AS1 silencing did not impact the nascent ALKBH3 pre‐mRNA level (Figure 4E), which indicated that ALKBH3‐AS1 might affect the expression of ALKBH3 at the post‐transcription level. Next, we compared the stability of ALKBH3 mRNA in ALKBH3‐AS1 knockdown and control HCC cells. With the transcriptional inhibitor actinomycin D treatment, the half‐life of ALKBH3 mRNA in HCC cells with ALKBH3‐AS1 knockdown was shorter than in control cells (Figure 4F). These results verify that ALKBH3‐AS1 maintains the stability of ALKBH3 mRNA in HCC cells.

### 3.4 ALKBH3‐AS1 facilitates HCC cell proliferation and invasion via ALKBH3

**FIGURE 5:**
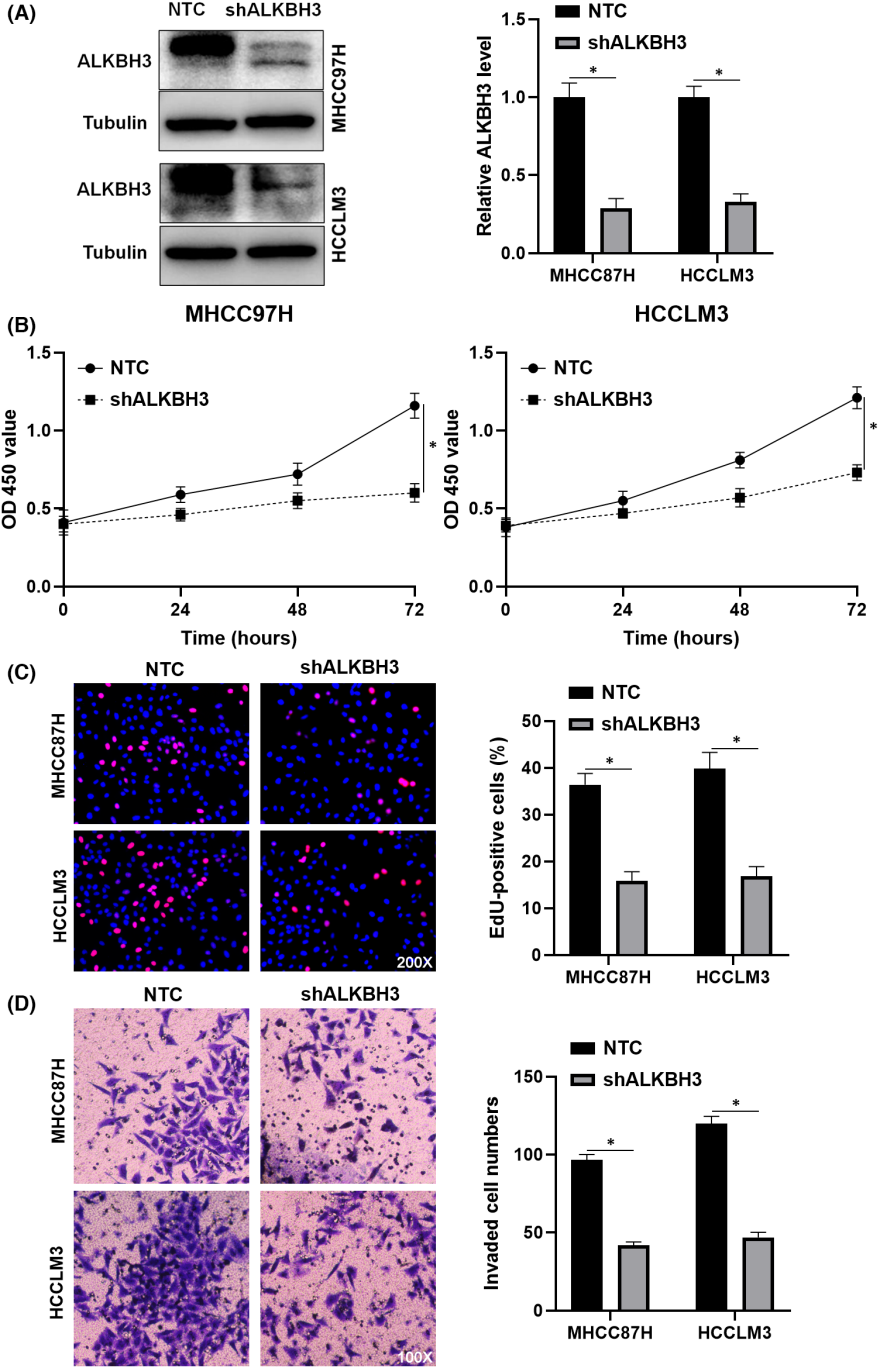
ALKBH3 knockdown inhibits HCC cell invasion and proliferation. (A) NTC or ALKBH3 shRNA (shALKBH3) transfection was performed in HCCLM3 and MHCC97H cells. Transfected HCC cells were measured by immunoblotting for the ALKBH3 level. (B–D) CCK‐8, EdU and transwell assays were carried out to investigate the proliferation and invasion of ALKBH3 knockdown and control HCC cells. *p < 0.05

**FIGURE 6:**
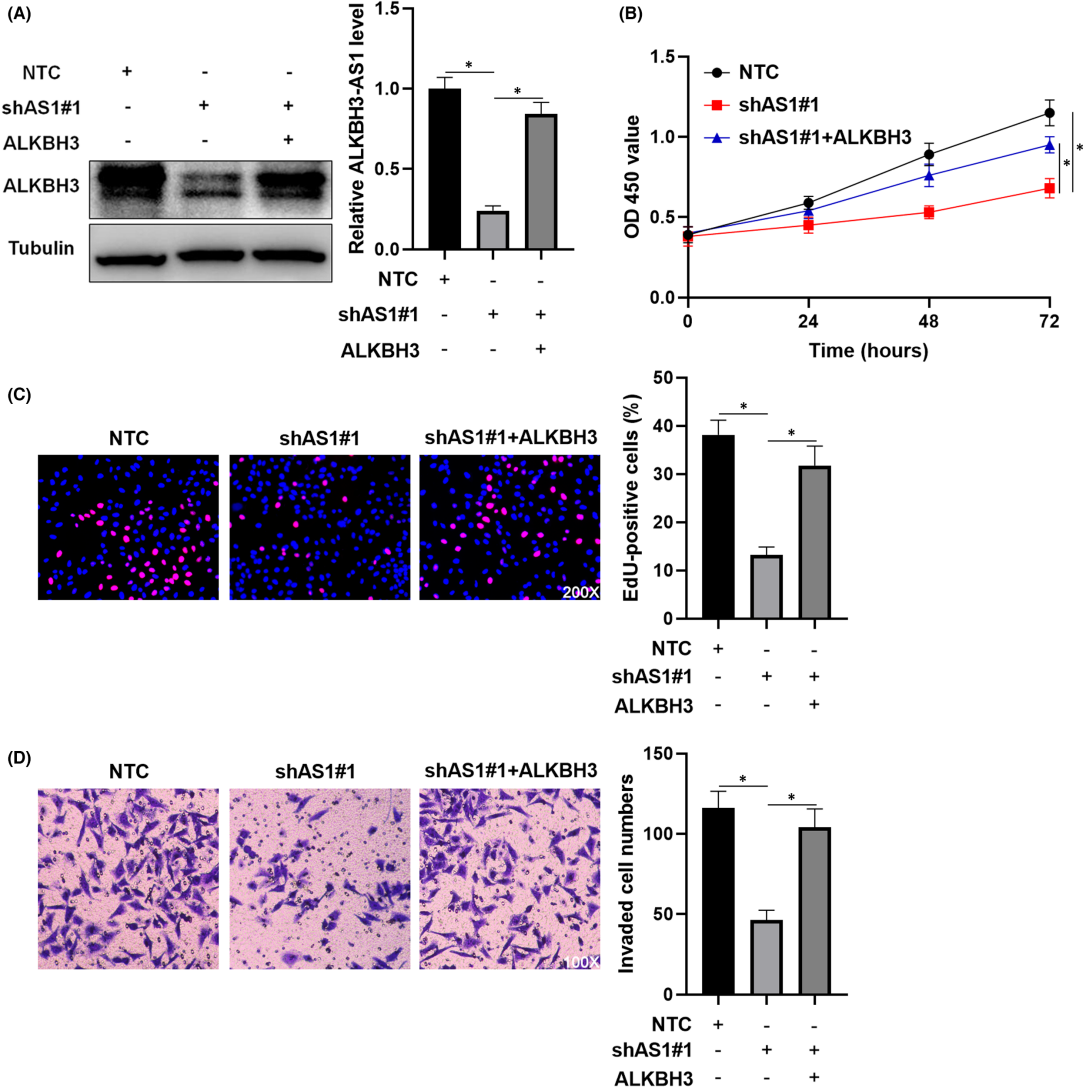
Restoring ALKBH3 expression attenuates the impact of ALKBH3‐AS1 knockdown on HCCLM3 cells. (A) HCCLM3 cells that were transduced with NTC, shAS1#1 or shAS1#1 + ALKBH3 were assessed by immunoblotting for the ALKBH3 level. (B–D) CCK‐8, EdU and transwell assays were used for exploring the proliferation and invasion of HCCLM3 cells with corresponding vector transfection. *p < 0.05

A previous study has confirmed that ALKBH3 promotes HCC growth.^20^ Here, we further demonstrated that ALKBH3 knockdown remarkably repressed the proliferation and invasion of HCC cells (*p* < 0.05, Figure 5A–D). Next, we restored ALKBH3 expression in ALKBH3‐AS1 knockdown HCCLM3 cells (*p* < 0.05, Figure 6A). CCK‐8, EdU and transwell assays revealed that ALKBH3 attenuated ALKBH3‐AS1 knockdown's inhibitory effects on HCCLM3 cell proliferation and invasion (*p* < 0.05, Figure 6B–D). Thus, ALKBH3‐AS1 functions as a pro‐HCC factor via ALKBH3.

### 3.5 Hypoxia induces ALKBH3‐AS1 expression via HIF‐1α

**FIGURE 7:**
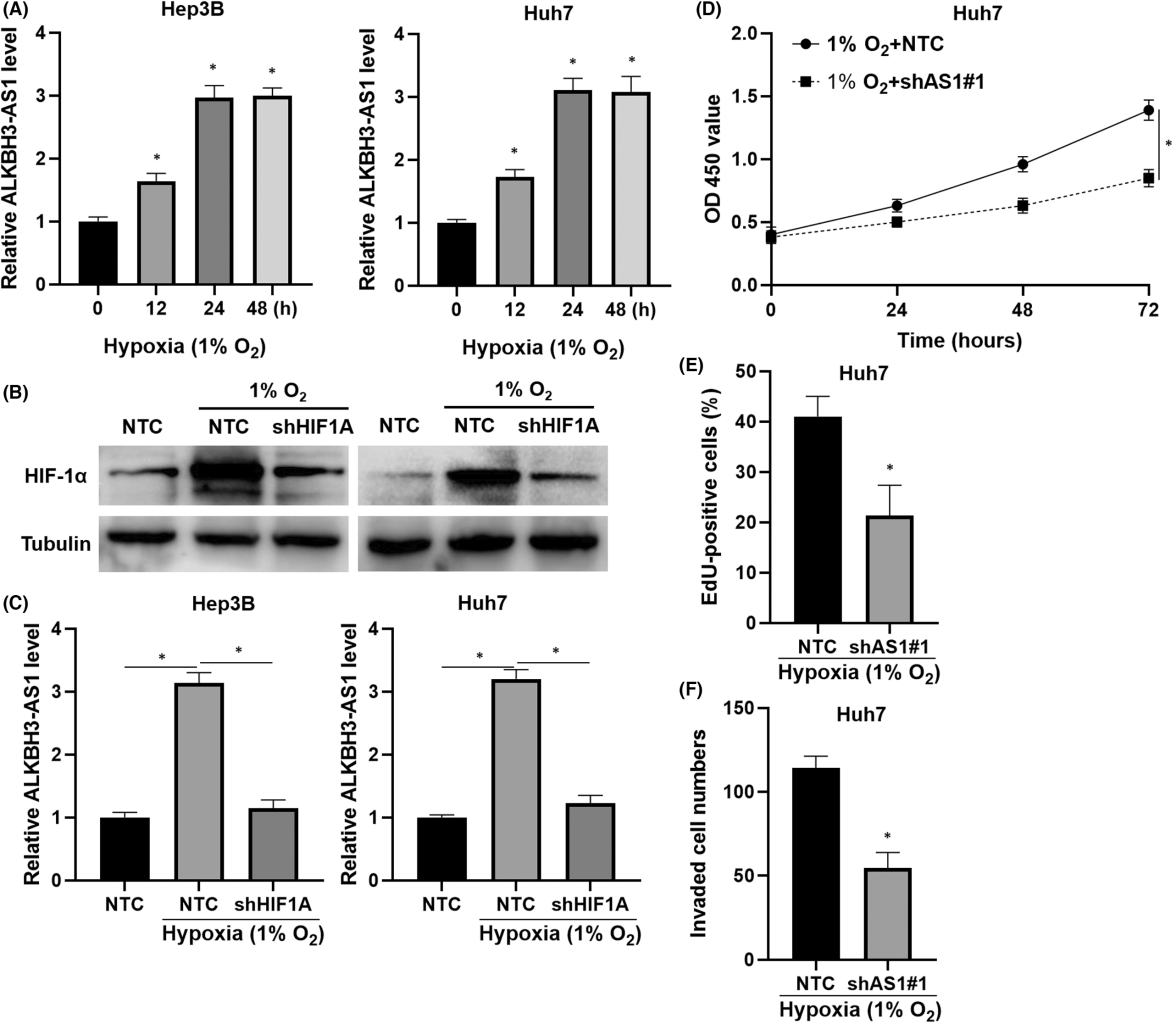
HIF‐1α transcriptionally regulates ALKBH3‐AS1 expression. (A) ALKBH3‐AS1 expression in HCC cells cultured in hypoxia conditions (1% O_2_) for different times. (B) The HIF‐1α shRNA (shHIF1A) and NTC were transfected into HCC cells, respectively. The HIF‐1α level was measured by Western blotting in HCC cells. (C) The ALKBH3‐AS1 level in HCC cells under different conditions was detected by RT‐qPCR. (D–F) CCK‐8, EdU and transwell assays were used for exploring hypoxic Huh7 cell invasion and proliferation after ALKBH3‐AS1 knockdown. *p < 0.05

Then, we intended to discover the potential upstream regulatory mechanism of ALKBH3‐AS1. The lncRNA microarray data (GSE155505) published in our previous study indicated an upregulated level of ALKBH3‐AS1 in Hep3B cells under hypoxia conditions.^21^ Further experiments confirmed that hypoxia upregulated ALKBH3‐AS1 levels in Hep3B and Huh7 cells (*p* < 0.05, Figure 7A). Otherwise, HIFα activator (DMOG) treatment also prominently increased ALKBH3‐AS1 expression in HCC cells (*p* < 0.05, Figure S3). HIF‐1α acts as a main transcription factor in the hypoxic microenvironment.^22^ We downregulated HIF‐1α in hypoxic HCC cells via shRNA transfection (Figure 7B). The RT‐qPCR analysis found that hypoxia‐enhanced ALKBH3‐AS1 expression was prominently abolished by HIF‐1α knockdown in HCC cells (*p* < 0.05, Figure 7C). Notably, TCGA‐LIHC data analysis using the GEPIA platform^18^ revealed that HIF‐1α mRNA was positively correlated with both ALKBH3‐AS1 and ALKBH3 mRNA in HCC tissues (*p* < 0.05, Figure S4). ALKBH3‐AS1 knockdown markedly attenuated cell invasion and proliferation in hypoxic Huh7 cells (*p* < 0.05, Figure 7D–F). Therefore, we identify ALKBH3‐AS1 as a HIF‐1α target gene.

## 4 DISCUSSION

The aberrant levels of lncRNAs have been observed in HCC, which can be used as diagnostic and prognostic markers.^5^ LncRNA‐ATB in exosomes arising from HCC patients' serum was associated with advanced tumour stage, portal vein thrombosis and poor prognosis.^23^ LINC00853, which is expressed in serum small extracellular vesicles, is identified as a powerful marker for early HCC diagnosis.^24^ The highly expressed lncRNA MCM3AP‐AS1 has been found in HCC tissues and associated with advanced tumour stage, poor tumour differentiation, large tumour and decreased survival.^9^ Here, we firstly verified the upregulation of ALKBH3‐AS1 in HCC. Clinical data analysis revealed that ALKBH3‐AS1 was expressed at a higher level in HCC samples from patients with large tumours, advanced tumour stages and venous infiltration. The high level of ALKBH3‐AS1 conferred poor overall survival and disease‐free survival as determined by TCGA‐LIHC data analysis. The above results suggest that ALKBH3‐AS1 may be used as a novel marker for predicting the poor prognosis of HCC.

LncRNAs participate in the occurrence and development of HCC by functioning as oncogenes or tumour suppressors.^5^ Lnc‐UCID enhances HCC cell proliferation by promoting G1/S transition.^25^ LINC01138 functions as an oncogene by contributing to HCC growth and metastasis.^26^ On the contrary, PSTAR and LINC01554 are tumour‐suppressive factors in HCC.^27^ Here, we found that ALKBH3‐AS1 knockdown repressed and ALKBH3‐AS1 overexpression increased the invasion and proliferation of HCC cells. ALKBH3‐AS1 silencing also reduced HCC growth in nude mice. Thus, ALKBH3‐AS1 is a pro‐HCC factor. LncRNAs are implicated in HCC occurrence and progression through various mechanisms.^6^ Previous evidence demonstrates that antisense lncRNAs modulate their neighbouring genes' expression.^19^ LINC01134 recruits chromatin‐modulating factors WDR5 and GADD45A to the promoter of SATB2, thereby enhancing SATB2 transcription and conferring the tumorigenesis and progression of colorectal cancer (CRC).^28^ LncRNA FOXC2‐AS1 forms an RNA duplex with FOXC2 mRNA to increase its stability and facilitate CRC progression by activating the Ca^2+^/FAK pathway.^17^ ALKBH3 is the neighbouring gene of ALKBH3‐AS1. A previous study has reported that ALKBH3 expression is increased in HCC and associated with poor clinical outcomes.^20^ ALKBH3 silencing restrained HCC cell proliferation and restricted tumour growth in nude mice.^20^ In this study, we further confirmed the overexpression of ALKBH3 in HCC via analysing TCGA‐LIHC data. The positive correlation between ALKBH3‐AS1 and ALKBH3 mRNA levels was confirmed in HCC tissues. We found that ALKBH3‐AS1 knockdown had no impact on the nascent ALKBH3 pre‐mRNA level in HCC cells. Thus, ALKBH3‐AS1 did not affect ALKBH3 expression at the transcription level. The half‐life of ALKBH3 mRNA in HCC cells was reduced after ALKBH3‐AS1 knockdown, indicating that ALKBH3‐AS1 maintained the stability of ALKBH3 mRNA. Notably, ALKBH3 silencing presented similar effects with ALKBH3‐AS1 knockdown in HCC cells. ALKBH3 restoration significantly attenuated the effects of ALKBH3‐AS1 knockdown on HCC cell invasion and proliferation. These data suggest that ALKBH3‐AS1 promotes HCC cells' malignant behaviours by enhancing ALKBH3 mRNA stability.

In the hypoxic microenvironment, HIF‐α is accumulated due to protein degradation inhibition and transcriptionally activated target genes' expression to promote HCC progression.^29^ Peptidylarginine deiminase 4 (PADI4) is transcriptionally regulated by HIFs and is involved in HIFs transcription activity by inducing histone citrullination.^30^ LncRNA TM4SF1‐AS1 is a hypoxia‐responsive gene and is modulated by HIF‐1α at the transcription level in HCC.^11^ In this study, both hypoxia and DMOG treatment upregulated the ALKBH3‐AS1 level, which was attenuated by HIF‐1α silencing in HCC cells. Otherwise, ALKBH3‐AS1 knockdown repressed the invasion and proliferation of hypoxic HCC cells. Our data reveal that HIF‐1α‐activated ALKBH3‐AS1 participates in hypoxia‐enhanced HCC progression.

In summary, our findings demonstrated that ALKBH3‐AS1 was transcriptionally regulated by HIF‐1α in the hypoxic microenvironment. ALKBH3‐AS1 acted as an oncogene to promote HCC cells' malignant behaviours by enhancing ALKBH3 mRNA stability. This project will provide new targets for the development of anti‐HCC drugs.

## AUTHOR CONTRIBUTIONS

**Qiliang Lu:** Data curation (equal); investigation (equal); methodology (equal); visualization (equal); writing – original draft (equal). **Hao Wang:** Data curation (equal); investigation (equal); methodology (equal); visualization (equal). **Xiangxiang Lei:** Data curation (equal); investigation (equal); methodology (equal). **Qiancheng Ma:** Data curation (equal); investigation (equal); methodology (equal). **Jie Zhao:** Data curation (equal); investigation (equal); methodology (equal). **Wen Sun:** Data curation (equal); investigation (equal); methodology (equal). **Cheng Guo:** Resources (equal). **Dongsheng Huang:** Conceptualization (equal); funding acquisition (equal); project administration (equal); supervision (equal). **Qiuran Xu:** Conceptualization (equal); funding acquisition (equal); project administration (equal); supervision (equal); writing – original draft (equal).

## CONFLICT OF INTEREST

The authors declare that they have no conflict of interests.

## Supporting information


Figure S1

Figure S2

Figure S3

Figure S4

Table S1

## Data Availability

The data that support the findings of this study are available from the corresponding author upon reasonable request.
