# Supplementary figures and images for: LncRNA ALKBH3‐AS1 enhances ALKBH3 mRNA stability to promote hepatocellular carcinoma cell proliferation and invasion
Source: J Cell Mol Med. 2022 Sep 13;26(20):5292–302. doi: 10.1111/jcmm.17558 (PMC9575106; doi:10.1111/jcmm.17558)

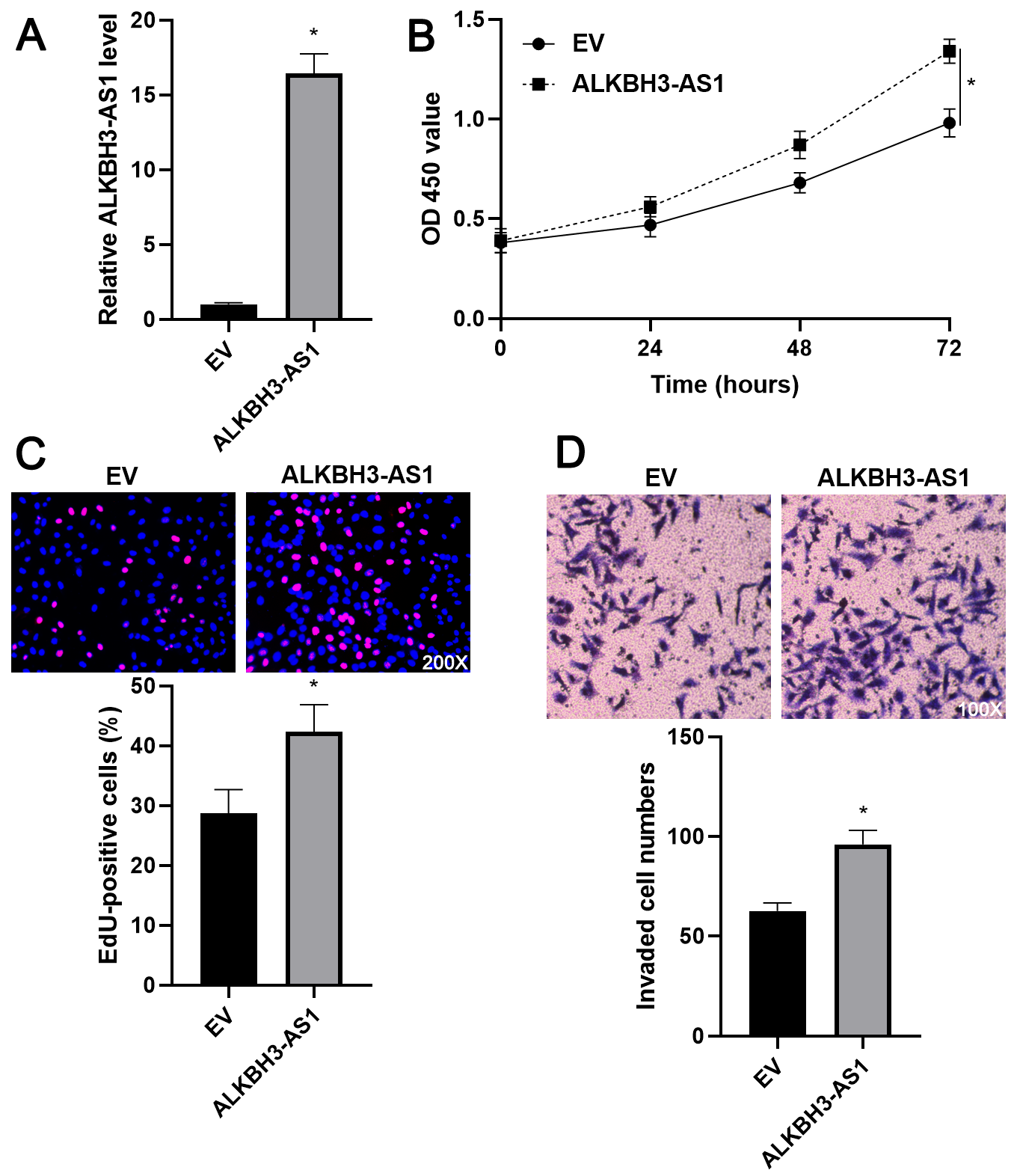

Supplement: Supplementary file 1 — Figure S1 [file JCMM-26-5292-s001.tif]

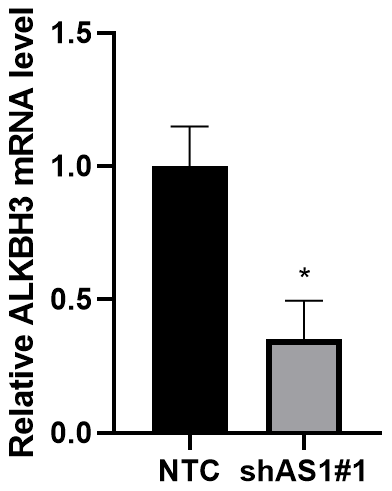

Supplement: Supplementary file 2 — Figure S2 [file JCMM-26-5292-s005.tif]

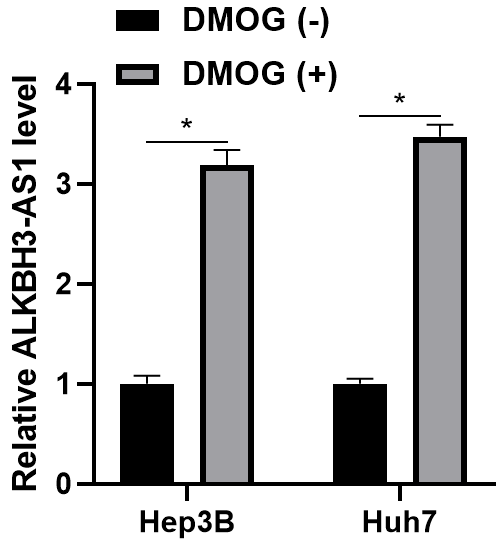

Supplement: Supplementary file 3 — Figure S3 [file JCMM-26-5292-s004.tif]

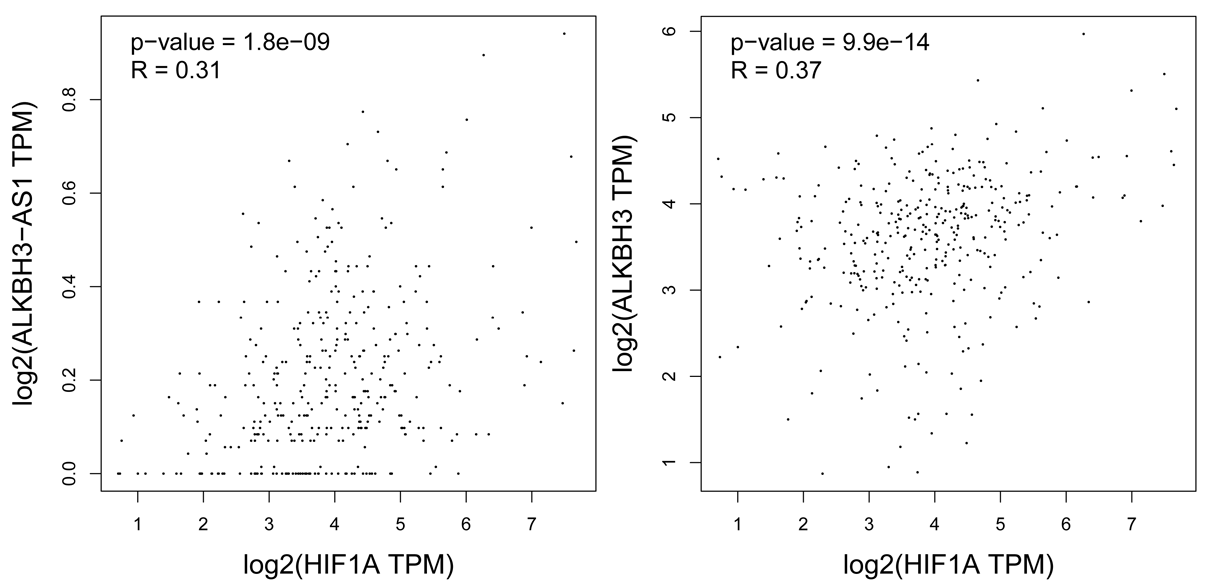

Supplement: Supplementary file 4 — Figure S4 [file JCMM-26-5292-s002.tif]
