# Supplementary material for: LncRNA ALKBH3‐AS1 enhances ALKBH3 mRNA stability to promote hepatocellular carcinoma cell proliferation and invasion
Source: J Cell Mol Med. 2022 Sep 13;26(20):5292–302. doi: 10.1111/jcmm.17558 (PMC9575106; doi:10.1111/jcmm.17558)
Supplement: Supplementary file 5 — Table S1 [file JCMM-26-5292-s003.docx]

**Supplementary Table 1.** Relationship between ALKBH3-AS1 expression and clinicopathologic parameters of patients with hepatocellular carcinoma

| Clinicopathologic parameters | | n=80 | ALKBH3-AS1 | | *P* |
| --- | --- | --- | --- | --- | --- |
|  |  |  | Low expression (n=40) | High expression (n=40) |  |
| Age (years) | <50 | 33 | 14 | 19 | 0.256 |
|  | ≥50 | 47 | 26 | 21 |  |
| Sex | Male | 67 | 33 | 34 | 0.762 |
|  | Female | 13 | 7 | 6 |  |
| HBV infection | No | 13 | 8 | 5 | 0.363 |
|  | Yes | 67 | 32 | 35 |  |
| Serum AFP level (ng/mL) | <20 | 14 | 10 | 4 | 0.078 |
|  | ≥20 | 66 | 30 | 36 |  |
| Tumor size (cm) | <5 | 29 | 20 | 9 | 0.011^*^ |
|  | ≥5 | 51 | 20 | 31 |  |
| No. of tumor nodules | 1 | 65 | 35 | 30 | 0.152 |
|  | ≥2 | 15 | 5 | 10 |  |
| Cirrhosis | No | 12 | 4 | 8 | 0.210 |
|  | Yes | 68 | 36 | 32 |  |
| Venous infiltration | No | 44 | 28 | 16 | 0.007^*^ |
|  | Yes | 36 | 12 | 24 |  |
| Tumor differentiation | I+II | 53 | 30 | 23 | 0.098 |
|  | III+IV | 27 | 10 | 17 |  |
| TNM stage | I | 32 | 22 | 10 | 0.006^*^ |
|  | II+III | 48 | 18 | 30 |  |
| BCLC stage | 0+A | 59 | 33 | 26 | 0.075 |
|  | B+C | 21 | 7 | 14 |  |

HBV, hepatitis B virus; AFP, alpha-fetoprotein; TNM, tumor-node-metastasis; BCLC, Barcelona Clinic Liver Cancer.

The “low” or “high” expression of ALKBH3-AS1 level was defined according to the cut-off value, which was defined as the median value of the cohort of patients tested.

^*^Statistically significant.
